# Three-dimensional printed polylactic acid and hydroxyapatite composite scaffold with urine-derived stem cells as a treatment for bone defects

**DOI:** 10.1007/s10856-022-06686-z

**Published:** 2022-10-03

**Authors:** Xiang Zhang, Jia-Lei Chen, Fei Xing, Xin Duan

**Affiliations:** 1 Department of Orthopaedics, West China Hospital, Sichuan University, No. 37 Guoxue Lane, Chengdu, 610041 Sichuan China; 2 Department of Orthopedics, Ganzi Tibetan Autonomous Prefecture People’s Hospital, Ganzi Prefecture, 626700 Sichuan China

## Abstract

Bone defects still pose various challenges in osteology. As one treatment method for bone defects, tissue engineering requires biomaterials with good biocompatibility and stem cells with good differentiation. This study aimed to fabricate a 3D-printed polylactic acid and hydroxyapatite (PLA/HA) composite scaffold with urine-derived stem cells (USCs) to study its therapeutic effect in a rat model of skull defects. USCs were isolated and extracted from the urine of healthy adult males and inoculated onto PLA/HA and PLA scaffolds fabricated by 3D printing technology. A total of 36 skull defect models in eighteen Sprague–Dawley rats were randomly divided into a control group (no treatment of the defects), PLA group (treated with PLA scaffolds with USCs), and PLA/HA group (treated with PLA/HA scaffolds with USCs). The therapeutic efficacy was evaluated by real-time PCR, microcomputed tomography (micro-CT), and immunohistochemistry at 4, 8, and 12 weeks. We found that the PLA/HA scaffold loaded with USCs effectively promoted new bone regeneration in the defect area. CT images showed that in the PLA/HA group, the defect area was almost entirely covered by newly formed bone (coverage of 96.7 ± 1.6%), and the coverage was greater than that in the PLA group (coverage of 74.6 ± 1.9%) at 12 weeks. Histology and immunohistochemical staining showed the highest new bone formation on the PLA/HA scaffolds containing USCs in the defect site at 12 weeks. These findings demonstrate the broad application prospects of PLA/HA scaffolds with USCs in bone tissue engineering.

**Figure:**
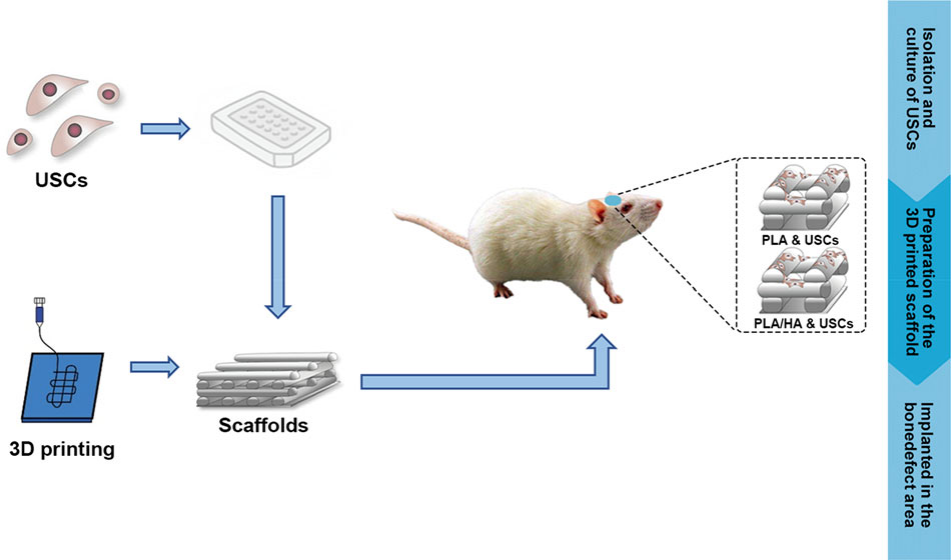
Graphical abstract

Graphical abstract

## Introduction

Bone defects caused by trauma, osteomyelitis, and tumor resections are common in clinical practice [1]. Millions of bone transplants are performed each year. Therefore, bone defect repair has long been the focus of orthopedic research [2]. Traditional treatments for bone defects include autogenous bone grafts, allografts, and bone graft substitutes. With osteoblasts, extracellular matrix, and bioactive factors, the transplanted bone tissue mediates fracture healing and enhances mechanical stress at the defect site. However, the traditional treatment for bone defects has apparent limitations due to immune rejection, the risk of disease transmission, poor bone histocompatibility, and other problems [3]. Improved treatments for bone defects are needed, and the emergence of tissue engineering and 3D printing technology has provided a new method and direction [4].

Urine-derived stem cells (USCs) are pluripotent adult stem cells whose biological properties resemble those of mesenchymal stromal cells (MSCs). USCs play a crucial role in promoting tissue regeneration and regulating immunity by secreting various cytokines [5]. Compared with other stem cells, USCs have the potential to differentiate into bone, cartilage, and adipose tissue, are easily obtained; can be collected without injury; and are highly practicable [6–8]. USCs have demonstrated potent therapeutic effects in animal models of the urinary system, nerve tissue, and skeletal muscle tissue injury. As stem cells, USCs are also used for urinary system reconstruction and bone tissue engineering [9–12]. Therefore, a therapeutic strategy based on USCs show promise for bone defect repair.

When applied to medical research, 3D printing technology has the advantages of short production cycles and low processing costs. With the help of computer-aided design (CAD), desirable engineered bone scaffolds are easy to acquire, which solves the problem of preparing scaffolds with unique structures and distribution patterns [13]. Compared with traditional technology, 3D printing can control the structure of biological scaffolds from the macro to the microscale, produce different types of tissue engineering scaffolds and achieve precision medicine [14]. As a standard technology in 3D printing, fused-deposition modeling (FDM) technology requires less equipment, has a more straightforward operation, and has a lower price than other technologies. By fixing the raw materials into filament-like materials and melting the filament-like materials through nozzle heating, the target bracket can be printed on the operating plate [15, 16].

Selecting a suitable scaffold is an essential step in bone tissue engineering. Due to its biocompatibility and degradability, polylactic acid (PLA), one of the commonly used synthetic polyester materials for preparing scaffolds, meets the requirements of bone tissue engineering. However, the degradation of polylactic acid under normal physiological conditions can generate acidic substances and cause inflammation [17]. Zhang et al. found that the preparation of composite scaffolds by mixing hydroxyapatite (HA) and PLA resulted in good biocompatibility and bioactivity with no differences in inflammatory reactions [18, 19]. Previous studies have demonstrated the mechanical properties of PLA enhanced with HA, and the applicability of PLA/HA scaffolds in tissue engineering [20]. However, scaffolds with an acceptable tolerance have not been applied to the development of USCs for bone regeneration, and their performance in tissue engineering has not been assessed.

Thus, we prepared a 3D-printed PLA/HA composite scaffold seeded with USCs isolated from healthy adult male urine. Then, we investigated its feasibility for bone defect repair based on the results of osteogenic induction in a rat skull defect model.

## Materials and methods

### Isolation and culture of USCs

The age of the urine donor is an essential factor in the application potential of USCs in bone tissue engineering. The microenvironment of aged donors may not be suitable for cell proliferation and differentiation; in addition, USCs from elderly donors tend to be senescent [21]. Thus, we collected urine from young volunteers aged 20–30 years at the Physical Examination Center of West China Hospital of Sichuan University. One milliliter of 1% penicillin–streptomycin (Procell Life Science & Technology, China) was added to each centrifuge tube containing the collected urine. After centrifugation at 1500 rpm for 15 min and flushed with phosphate-buffered saline (PBS) (Sigma-Aldrich, USA), the collected cell precipitates were added to six-well plates, with 2 ml of Dulbecco’s modified Eagle’s medium (DMEM) (Thermo Fisher Scientific, China) with 2% (vol/vol) fetal bovine serum (FBS) (Thermo Fisher Scientific, China), 10 ng/ml human epidermal growth factor (hEGF) (Thermo Fisher Scientific, China), 2 ng/ml platelet-derived growth factor (Thermo Fisher Scientific, China), 1 ng/ml transforming growth factor-β (TGF-β) (Thermo Fisher Scientific, China), 0.5 μM cortisol (hydrocortisone) (Thermo Fisher Scientific, China), 25 μg/ml insulin (Thermo Fisher Scientific, China), 20 μg/ml transferrin (Thermo Fisher Scientific, China), 549 ng/ml adrenaline, L-Glu and antibiotics (Thermo Fisher Scientific, China). The DMEM was replaced every 3 days. Subsequently, the cells were incubated at 37 °C and 5% CO_2_ for further experiments. After the cells reached 80–90% confluence, the cell pellets were flushed with PBS, and then, prepared trypsin solution (Sigma-Aldrich, USA) was added to digest the adherent cells. After centrifugation at 1500 rpm for 5 min, the cell pellets were cultured in a 25 T breathable culture flask (Corning Costar, USA) with a CO_2_ concentration of 5% at 37 °C. Cells were frozen and stored in liquid nitrogen after the subculture reached the fourth passage. Passage 4 cells USCs were used for our experiments.

### Identification of USCs

USCs are believed to express the same surface antigens as MSCs (CD29, CD44, CD54, CD73, CD90, and CD105) rather than hematopoietic stem cell markers (CD11b, CD14, CD19, CD31, CD34, CD45, and HLA-DR) [5, 7]. USCs were identified by flow cytometry. First, the cell concentration of passage 4 USCs was adjusted to 1 × 10^7^ cells/200 μl. The USCs were fixed with 4% paraformaldehyde (Sigma-Aldrich, USA). Then, high expression of CD73, CD90, and CD105 on the cell surface was used as a positive indicator of USCs, while low expression of CD34, CD45, MHCII, HLA-DR, and other stem cell surface markers was used as a negative indicator of USCs. After addition of the corresponding antibodies (Abcam, UK), USCs were incubated at 4 °C for 30 min. Finally, the cells were repeatedly washed with PBS and identified by flow cytometry.

USCs can differentiate into osteoblasts, chondroblasts, and adipoblasts in the appropriate environment and induction medium [5, 22]. Therefore, the osteogenic differentiation of passage 4 USCs was identified by alizarin red staining and verified by alkaline phosphatase staining. USCs were cultured in osteogenic medium for 3 weeks. After being fixed with 4% paraformaldehyde for 30 min, the cell slides were stained with Alizarin red dye (Sigma-Aldrich, USA) in an incubator at 37 °C for 30 min. The prepared samples were observed under an inverted microscope (IXplore Standard, Olympus, Japan). In addition, the adipogenic and chondrogenic differentiation of USCs was identified by oil red O staining (Sigma-Aldrich, USA) and Alcian blue staining (Sigma-Aldrich, USA), respectively.

### Preparation of the 3D printed PLA/HA composite scaffold

PLA (Sigma-Aldrich, USA) and HA (Sigma-Aldrich, USA) were weighed according to different mass ratios (9:1). PLA was dissolved in trichloromethane (Sigma-Aldrich, USA), and heated to 60 °C in a water bath. During the stirring process, HA powder was added and blended well until all ingredients were fully dissolved. After sonication in an ultrasonic oscillator (SHA-B, Guohua, China) for 15 min, PLA and HA were thoroughly stirred for 10 h with an electromagnetic agitator (NanBei Instrument, China) to ensure that HA was evenly dispersed in the PLA, as previously described [23]. After the drying process, the mixed ingredients were added to a 3D printer (CR3040, Chuangxiang Industrial, China) to make a solid wire with a diameter of 0.5 mm. The printing conditions were set as follows: the diameter of the printing needle was 0.5 mm; the movement speed of the needle was 0.5 mm/s; the printing temperature was set to 70 °C; and the pause between the two printing layers was 0.1 s. CAD software (AutoCAD2018, Autodesk, USA) was used to design the shape of the bracket (rectangular sheet with a length and width of 20 mm and thickness of 1.5 mm), and the porosity was determined to be approximately 65%, as described previously [24].

### Scanning electron microscopy (SEM), energy-dispersive X-ray spectroscopy (EDS), and mechanical property analysis

The general view of the 3D-printed PLA and PLA/HA composite scaffolds was observed, and their sizes were measured. The microscopic morphology of the 3D-printed PLA and PLA/HA composite scaffolds was observed by SEM (JSM-IT300HR, Shimadzu, Japan). EDS (Thermo Fisher Scientific, China) was used to determine the presence of HA in PLA/HA. A universal mechanical properties tester (*n* = 5 in each group) (Shimadzu, Japan) was used to measure the mechanical tensile strain of the two scaffolds (all samples were continuously exposed to a constant strain rate of 0.5 mm/min without preloading until maximum deformation was achieved).

### Porosity and in vitro degradation rates of scaffolds

The porosity of the scaffolds (M) was measured by the soaking method. We calculated the volume (V) and weighed the mass (M_0_) of the scaffold. The sample was immersed in absolute alcohol (Sigma-Aldrich, USA) for 24 h, and the pores of the scaffold were filled under pressure. The samples were removed and weighed (M_1_). The porosity of each scaffold was calculated as follows: M (%) = (M_1_ − M_0_)/V × 100% (M represents the porosity of each scaffold; M_0_ represents the initial weight of the scaffold; M_1_ represents the weight of scaffolds after being immersed; V represents the volume of the scaffold).

The general evaluation of the degradation rates of the scaffolds was performed in PBS and was named the weight loss method [25]. After being dried and weighed (W_0_), PLA and PLA/HA scaffolds (*n* = 4 in each group) were placed in PBS, replaced every week, and heated in a water bath at 37 °C. At each time point (1–8 weeks), samples were collected, dried, and weighed (W_1_). The degradation rate of the material was calculated as follows: W_T_ (%) = (W_0_ − W_1_)/W_0_ × 100% (W_T_ represents the degradation rate of the scaffold; W_0_ represents the initial weight of the scaffold; W_1_ represents the weight of the scaffold after drying).

### Viability and proliferation of USCs on scaffolds

Passage 4 USCs were carefully seeded on PLA and PLA/HA composite scaffolds (cell concentration:10^5^ cells/ml, *n* = 4 of each type). After being cultured in DMEM with high glucose for 3 days, the scaffolds were flushed and fixed with 2.5% glutaraldehyde solution (Sigma-Aldrich, USA). The scaffolds with USCs were stored in a vacuum freeze-dryer (Songyuan Huaxing, China) for 24 h. Then, the scaffolds underwent a process of metal spraying. All samples were evaluated by SEM for morphology and the adhesion of cells. Cell permeabilization was performed on scaffolds with a 0.5% Triton X-100 solution. Next, TRITC phalloidin (1:200, Sigma-Aldrich, USA) was added to the surface of the scaffolds, and the scaffolds were incubated for 30 min in a dark room. Then, DAPI solution (1:1000, Sigma-Aldrich, USA) was added to the surface of the scaffolds for nuclear counterstaining after a PBS rinse. The cytoskeleton was observed by confocal laser scanning microscopy (FV1000, Olympus, Japan) and immunofluorescent staining [TRITC excited/emitter filters (Ex/EM = 540/570 nm) and DAPI excited/emitter filters (Ex/EM = 364/454 nm)]. A CCK-8 kit (Abcam, UK) was used to examine USCs under scaffold leach liquor, which was acquired by immersing the scaffolds in DMEM for 24 h in a 37 °C incubator. USCs were seeded in 96-well plates and incubated for 24 h. Then, leaching liquor with 10% FBS and 1% penicillin-streptomycin was used as the cell culture medium. At different time points (1, 3, 5, and 7 days after the experiment), CCK-8 reagent and a full-wavelength microplate reader (Allsheng, China) were used to measure the absorbance of the soaking solution at 450 nm in the different groups.

### Quantitative RT–PCR analysis of osteogenic gene expression

Passage 4 USCs were added to osteogenic medium and divided into three groups (control, PLA, and PLA/HA) at specific points (Days 1, 3, and 7; three auxiliary wells at each time point). RNA concentration and purity were determined by a Nanodrop 2000 (Thermo Fisher Scientific, China) after total RNA extraction. cDNA was acquired by reverse transcription of the extracted RNA. Subsequently, the osteogenic differentiation potential of USCs on the scaffolds was examined by real-time PCR (*OCN*, *COLI*, *ALP*, and *Runx2*). (The primer sequences are shown in Table 1).

**Table 1:** The sequence of analyzed gene primer for real-time PCR

Gene	Primer
COL I	Forward: 5′-GGCCCTCAAGGTTTCCAAGG-3′
	Reverse: 5′-GGCCCTCAAGGTTTCCAAGG-3′
Runx2	Forward: 5′-CGTTCACTCCCATGACAAACA-3′
	Reverse: 5′-CGTTCACTCCCATGACAAACA-3′
OCN	Forward: 5′-CCCTGCACCTCATCCCTGA-3′
	Reverse: 5′-CCCTGCACCTCATCCCTGA-3′
ALP	Forward: 5′-GGCCTGTACCATACAAGCCC-3′
	Reverse: 5′-CCACGTAGACGAGGTAGTTGTG-3′

### Rat cranial defect model

Eighteen Sprague–Dawley rats (no sex limitation, aged 6–8 weeks, average body weight 300 g) were anesthetized by an intramuscular injection of 3% pentobarbital sodium (50 mg/kg). Circular defects with 5 mm diameters were drilled with an orthopedic ring drill (Wuyang, China) on both sides of the sagittal line. Buprenorphine hydrochloride (0.05 mg/kg) (Shafei, China) was intraperitoneally injected for analgesia, and 200,000 U benzylpenicillin sodium (North China Pharmaceutical Company, China) was intramuscularly injected to reduce inflammation. A total of 36 skull defects in 18 rats were randomly divided into the control group (*n* = 12, without any treatment); PLA group (*n* = 12, the defect area was implanted with the PLA scaffold combined with USCs); and PLA/HA group (*n* = 12, the defect area was implanted with the PLA/HA scaffold combined with USCs).

### Micro-CT, hematoxylin–eosin (HE) and Masson staining, and histological analysis

Six rats were sacrificed by administration of a lethal dose of pentobarbital sodium at different times (4, 8, and 12 weeks) after treatment, and their skulls were removed to observe bone regeneration in the defect area. Samples were imaged by micro-CT (vivaCT40, SCANCO Medical, Switzerland) with a resolution of 21 μm for scanning and imaging. Three-dimentional images of the models were obtained, and the newly formed bone area measurements were performed by ImageJ software (version 1.51k, NIH, USA) at each time point.

**Scheme 1:**
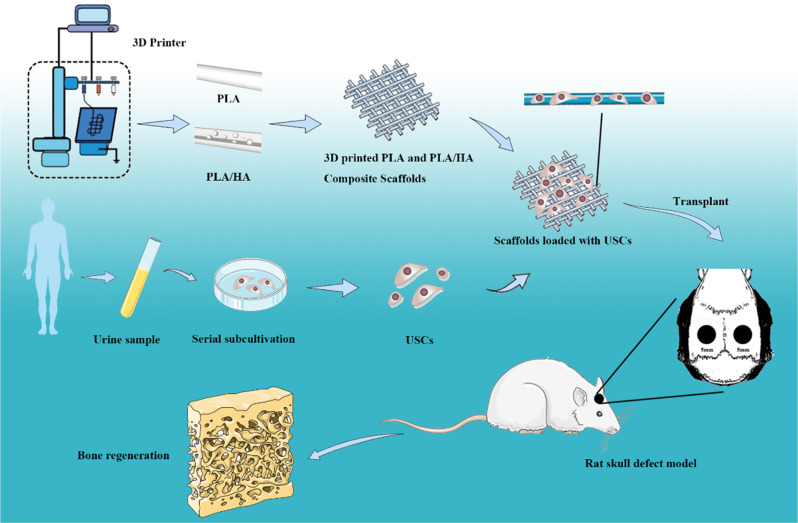
3D printed PLA and PLA/HA loaded with USCs as an artificial bone substitute for bone regeneration in a rat skull defect model

After being fixed in 4% paraformaldehyde, samples from the different groups were washed and placed in a 37 °C water bath for 7 days to remove calcium. Subsequently, the samples were embedded in paraffin (Sigma-Aldrich, USA) and sliced into 5-μm thick sections. Histological analysis was performed by using HE staining. Masson staining showed the formation of fibers in the newly formed tissue. In addition, the tissue sections were immunohistochemically stained (*COLI* and *OCN)*. All results were obtained using a fluorescence microscope (BX53, Olympus, Japan), and the newly formed bone area percentage in each group for staining was evaluated by ImageJ software (Scheme 1).

### Statistical analysis

GraphPad Prism (8.4.3) was used to analyze all experimental data. The data are presented as the mean ± SD, and ANOVA was used to analyze the differences between groups. *P* < 0.05 was considered statistically significant, and *P* < 0.01 was considered highly significant.

## Results

### Biological properties of USCs

**Fig. 1:**
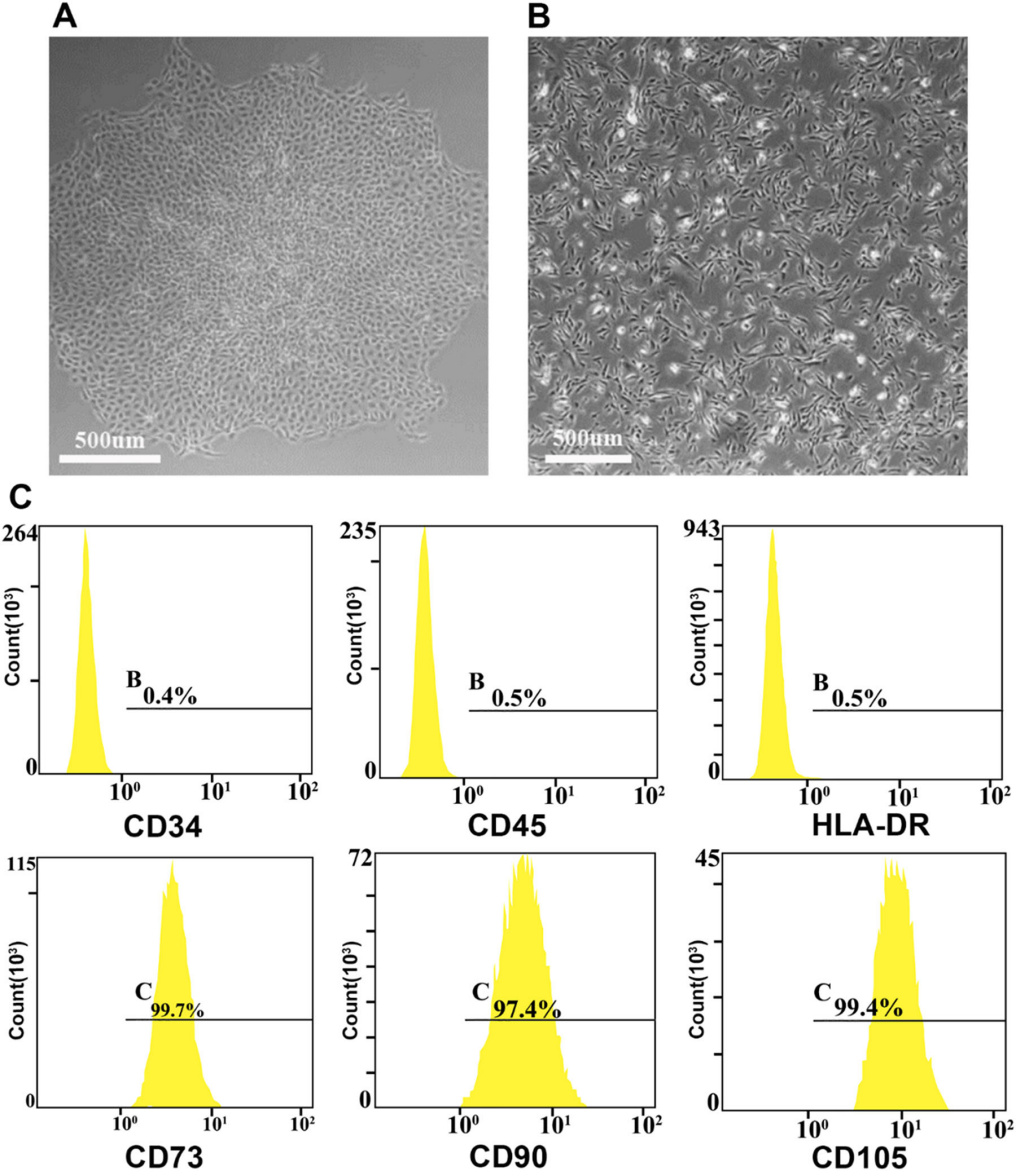
USCs were identified by microscopy and flow cytometry. **A** Morphology of primary USCs cultured for 2 weeks. **B** Morphology of fourth-generation USCs. **C** Flow cytometry results of negative (CD34, CS45, HLA-DR) and positive (CD73, CD90, CD105) antigens of fourth-generation USCs

USC colonies were visible under the microscope after 2 weeks of subculture (Fig. 1A). Passage 4 USCs showed slender shapes, and some polygonal shapes (Fig. 1B). Flow cytometry showed that surface antigens of blood cells (CD34, CD45, and MHC II HLA-DR) were not expressed or were expressed at low levels in USCs, while surface antigens of stem cells (CD73, CD90, and CD105) were highly expressed by over 95% of USCs (Fig. 1C), which was consistent with previous research [26]. These observations suggest that USCs can be effectively isolated from the urine of healthy adults by the isolation culture method used in this study.

**Fig. 2:**
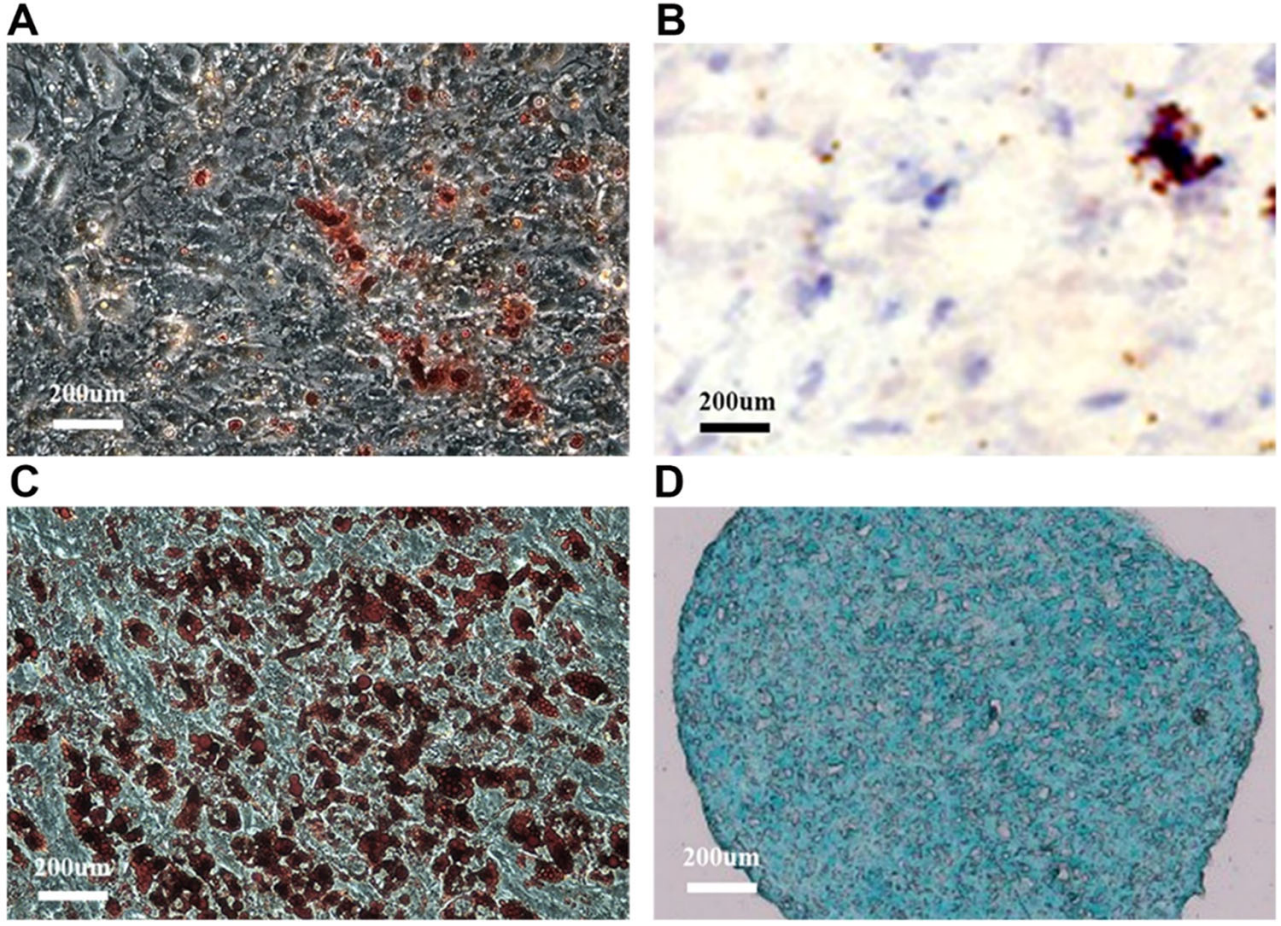
Differentiation analysis of USCs. **A** Alizarin red staining. **B** Alkaline phosphatase staining. **C** Oil red O lipid staining. **D** Alcian blue staining

Alizarin red staining showed that USCs became polygonal, and red-stained calcium nodules in the cell mass appeared after culture for 4 weeks (Fig. 2A). Positive alkaline phosphatase staining after osteogenic induction culture also indicated the formation of osteoblasts, which confirmed the osteogenic differentiation potential of USCs (Fig. 2B). Oil red O staining indicated that many red-stained lipid droplets formed in the cytoplasm after adipogenic induction (Fig. 2C). Alcian blue staining showed that abundant mucopolysaccharides appeared, which suggested that USCs possess a chondrogenic ability (Fig. 2D). Stem cells with multidirectional differentiation potential and reproductive activity are quality stem cells that meet the requirements of bone tissue engineering. The characteristics of USCs determine their excellent performance in bone defect repair.

### Characterization of the 3D-printed scaffolds

**Fig. 3:**
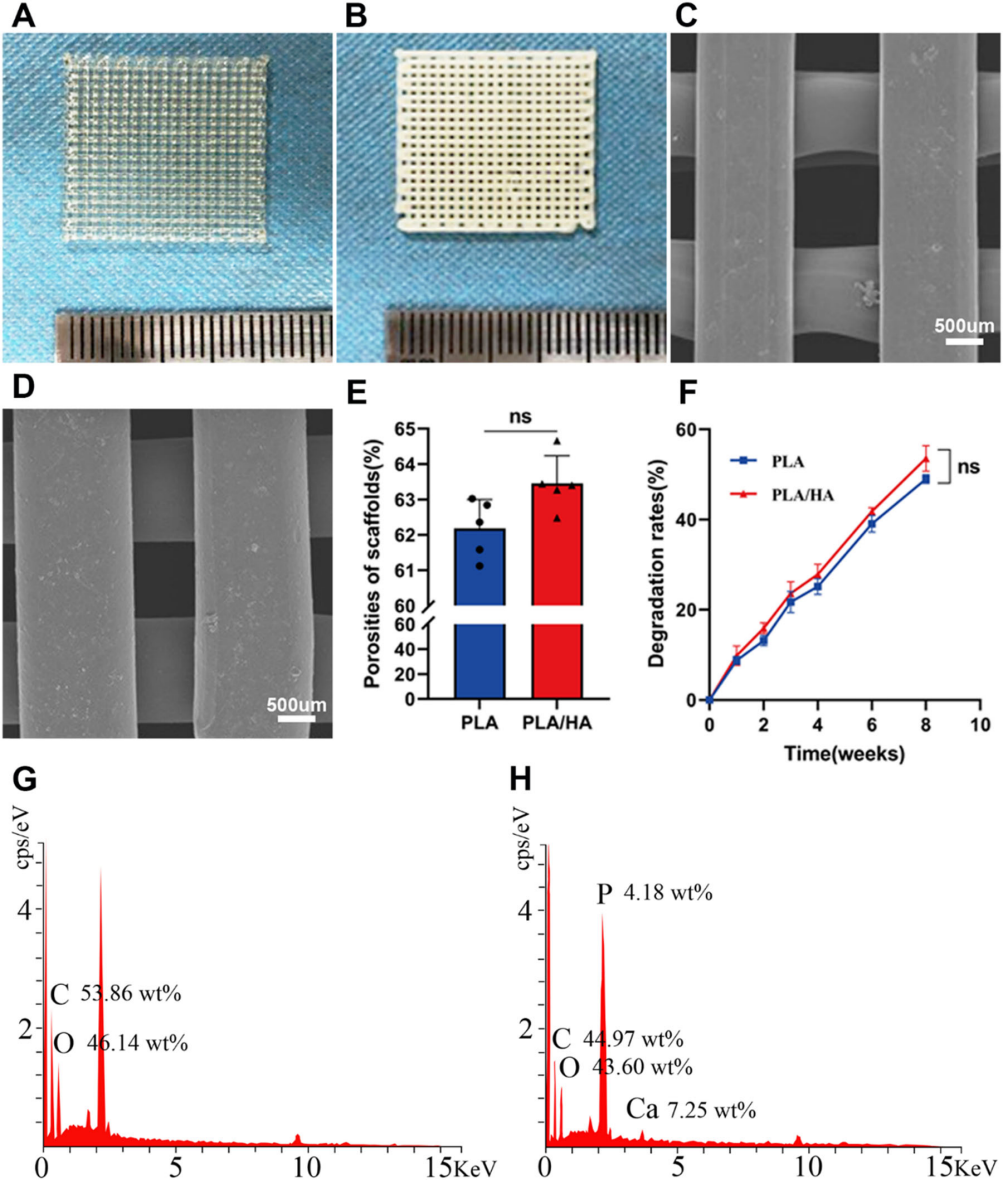
Preparation and characterization of the scaffolds. Overview of the PLA (**A**) and PLA/HA (**B**) scaffolds. SEM images of the PLA (**C**) and PLA/HA (**D**) scaffolds. The porosities (**E**) and degradation rates (**F**) of the PLA and PLA/HA scaffolds. **G**, **H** Electron dispersive X-ray spectroscopy (EDS) analysis indicates the presence of Ca and P in the PLA/HA scaffold

The printed scaffolds were 20 × 20 × 1.5 mm cubes, and the pore sizes of the scaffolds were between 300 and 500 µm. The PLA scaffold was almost transparent (Fig. 3A), while the PLA/HA composite scaffold was yellow (Fig. 3B). SEM revealed that these two scaffolds were neatly and evenly arranged, and the pores were connected to each other (Fig. 3C, D). The vast surface area was conducive to the adhesion and growth of USCs. In addition, the total porosity of these scaffolds was greater than 60%, which provided the necessary environment and ensured the conduction of nourishing substances for cell growth and the removal of metabolic waste (Fig. 3E). The degradation curves of these two scaffolds (Fig. 3F) suggested that the degradation speed of the PLA/HA scaffold was faster than that of the PLA scaffold. At 8 weeks, the degradation rates of the PLA and PLA/HA scaffolds were 49.0 ± 0.7% and 53.6 ± 2.2%, respectively (*P* ≥ 0.05).

The EDS results showed that in addition to three elements (C/H/O), the PLA/HA composite scaffold also contained Ca and P, which confirmed the chemical composition of HA, indicating that this scaffold was a combination of PLA and HA (Fig. 3G, H).

As expected, the mechanical properties of the PLA scaffold were significantly improved after the addition of HA (Table 2). The Young’s modulus of the PLA/HA scaffold (169.45 ± 30.46 MPa) was approximately 2 times that of the PLA scaffold (84.62 ± 12.45 MPa). The ultimate deformation strength of the PLA/HA scaffold (2.51 ± 0.21 MPa) was approximately 1.5 times that of the PLA scaffold (1.65 ± 0.19 MPa). These results indicated that the biomechanical force of the pure PLA scaffold was enhanced by the addition of HA.

**Table. 2:** Tested values of Young’s modulus and ultimate deformation strength of PLA and PLA/HA scaffolds

samples	Young’s modulus (MPa)	Ultimate deformation strength (MPa)
Cancellous Bone	425.19	75
PLA	84.62 ± 12.45	1.65 ± 0.19
PLA/HA	169.45 ± 30.46	2.51 ± 0.21

### Cytocompatibility of the printed scaffolds

**Fig. 4:**
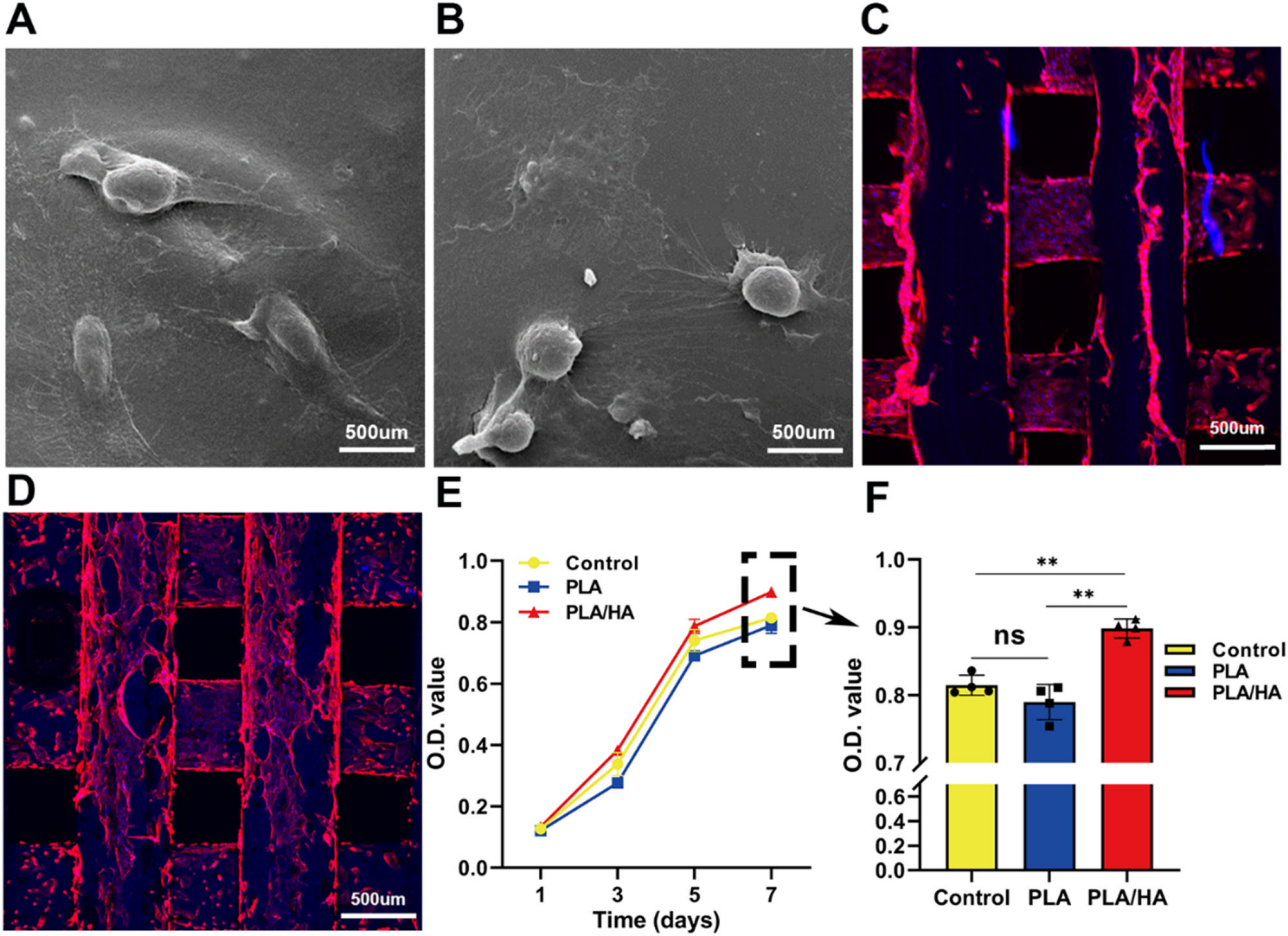
Cytocompatibility tests of the scaffolds. Electronic microscopy scans of USCs on PLA (**A**) and PLA/HA (**B**) scaffolds. The cytoskeleton was investigated by laser confocal microscopy (**C** PLA scaffold; **D** PLA/HA scaffold). **E**, **F** Growth of USCs in the control group and scaffold leaching liquor groups at each time point (1, 3, 5, 7 days after the experiment). *p < 0.05; **p < 0.01

After being cultured for 3 days on the scaffolds, USCs attached to the PLA scaffold and showed typical fibrous cell morphology, but only a small number of USCs were found by SEM (Fig. 4A). However, in the PLA/HA group, as shown in the enlarged image, groups of USCs stretched numerous filopodia and adhered tightly to the scaffold (Fig. 4B). Confocal microscopy showed that numerous filopodia around the surface of the USCs clung to the scaffold. On the PLA/HA composite scaffold, the USCs cytoskeleton was crosslinked into a network and spread evenly (Fig. 4D). However, USCs were unequally distributed over the surface of the PLA scaffold, and there were overlaps in the inner structure (Fig. 4C), which indicated that the PLA/HA composite scaffold may be more favorable for cell attachment and migration than the PLA scaffold. The CCK-8 assay showed that the cell growth curve in the scaffold leaching solution was similar to that of the growth medium group at each time point. (Fig. 4E). Besides, the PLA/HA scaffold exhibited the highest viability among three groups at day 7 (Fig. 4F) (P < 0.01). These results showed good cell proliferation and proliferation of USCs on the scaffolds.

### Expression of osteogenic genes in USCs on the scaffolds

**Fig. 5:**
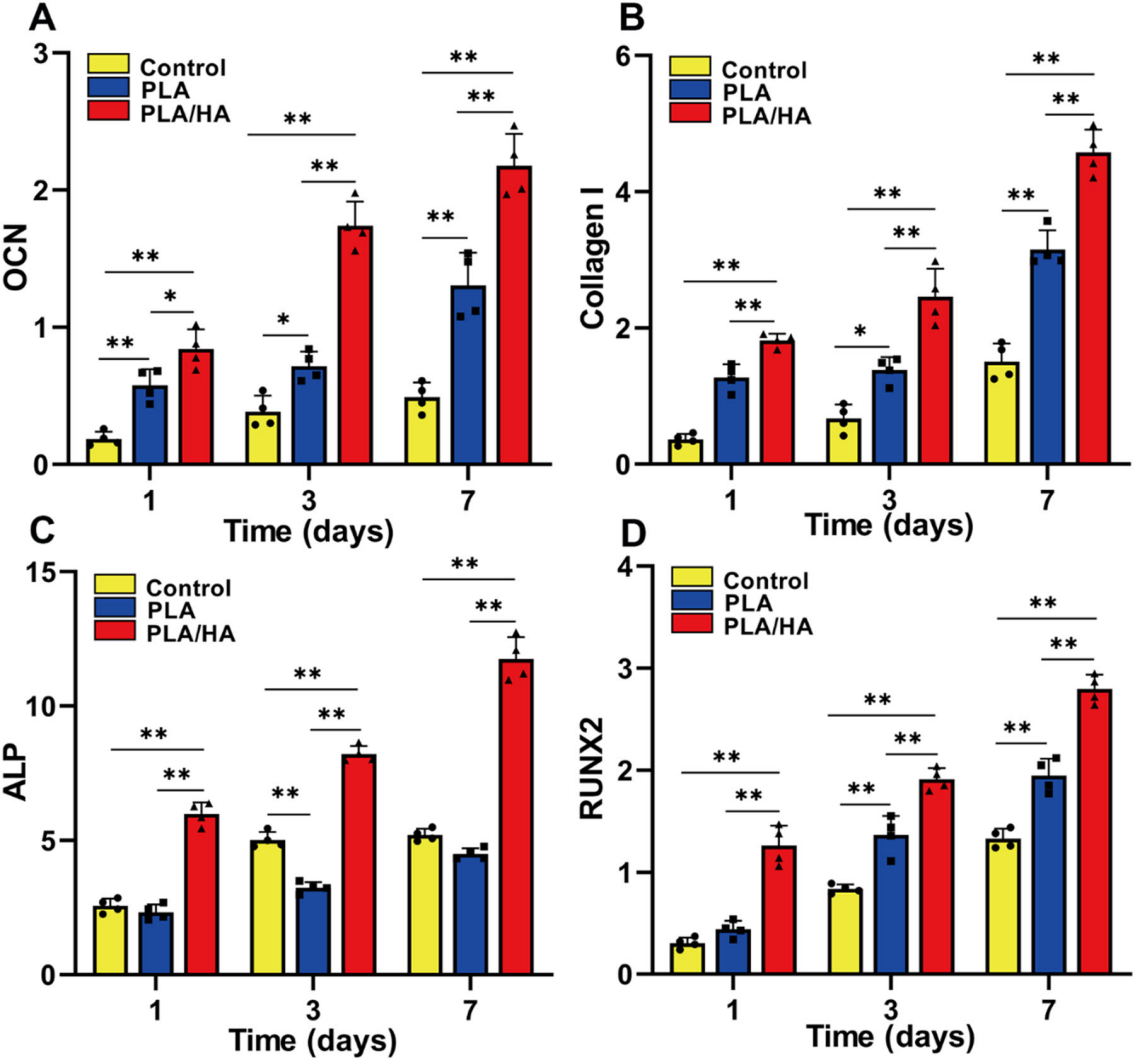
Relative mRNA expression levels of osteogenic genes in the different groups (Control, PLA and PLA/HA groups) at each time point (1, 3, 7 days after experiment). **A** *OCN*. **B** *COL I*. **C** *ALP*. **D** *RUNX2*. *p < 0.05; **p < 0.01

The expression of osteogenic genes (*OCN*, *COLI*, *ALP*, *Runx2*), which indicate bone remodeling, was examined by real-time quantitative PCR. The mRNA expression levels of *COLI*, *Runx2*, and *OCN* in the PLA and PLA/HA groups were significantly higher than those in the control group (Fig. 5A–D). The expression levels of *COLI*, *Runx2*, *OCN*, and *ALP* in the PLA/HA group were higher than those in the PLA group. However, the expression of *ALP* in the PLA group was lower than that in the control group.

### Evaluation of the repair of skull defects in rats

**Fig. 6:**
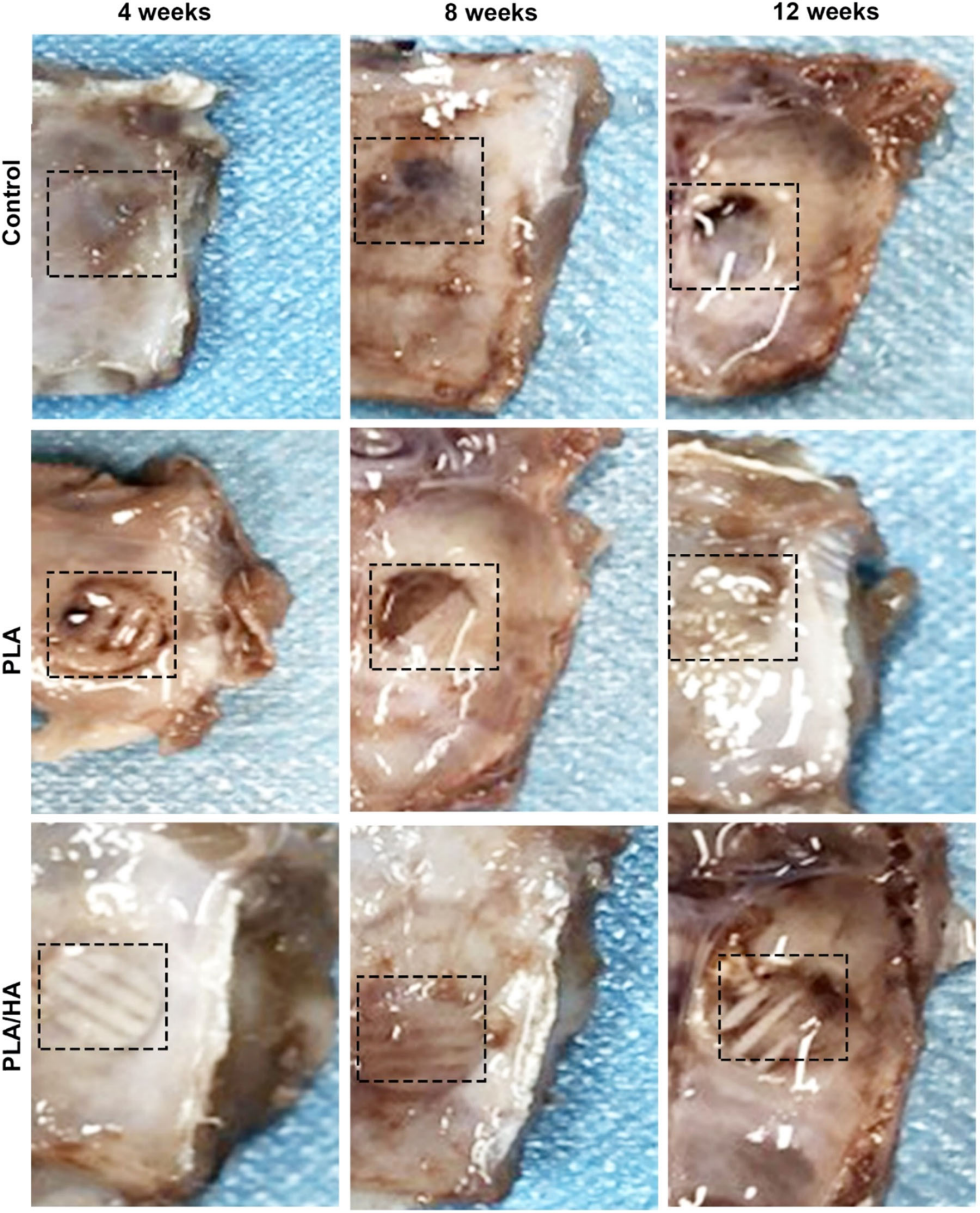
Photographs of skull defects in rats that received no treatment (control), PLA with USCs, and PLA/HA with USCs at each time point (4, 8, and 12 weeks after treatment)

Bone defect repair was examined in each group at 4, 8, and 12 weeks (Fig. 6). Bone regeneration in the samples with scaffold treatment was significantly more effective than that in the control group. Over time, the outlines of the scaffolds were vaguely visible, the boundaries between the scaffolds and defect edges were blurred, and the newly formed bone accumulated in the defect area. Bone defect repair in the PLA/HA group was more evident than that in the PLA-only group. However, the PLA/HA composite scaffold with USCs effectively promoted bone regeneration.

**Fig. 7:**
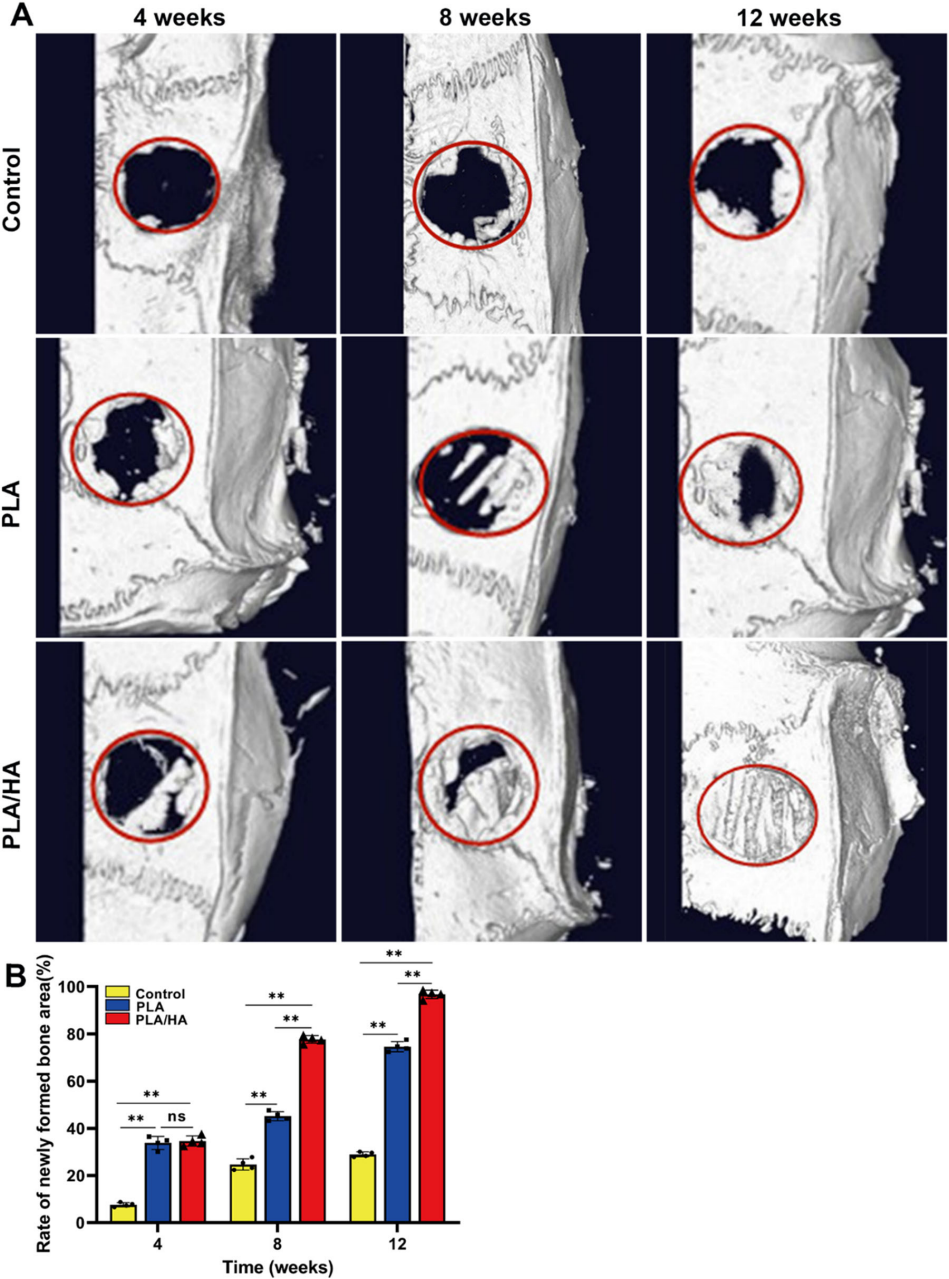
**A** Images reconstructed by micro-CT showing bone regeneration in the different groups (control, PLA, and PLA/HA) at each time point (4, 8 and 12 weeks after treatment). **B** The rate of newly formed bone tissue was measured by ImageJ software (version 1.51k, NIH, USA). *p < 0.05; **p < 0.01

Micro-CT results supported the general observations. Twelve weeks after treatment, there was little newly formed bone in the control group, with bone formation of only 29.0 ± 0.9%, while the PLA and PLA/HA groups showed progressively increasing amounts of newly formed bone, starting from the border of the defect area and scaffolds (Fig. 7A). Radiographic images of the newly formed bone area during the regeneration process with the PLA and PLA/HA scaffolds seeded with USCs indicated higher coverage of bone defects than that in the control group (96.7 ± 1.6% vs. 74.6 ± 1.9%), at 12 weeks after treatment (Fig. 7B), with a significant difference (*P* < 0.01).

**Fig. 8:**
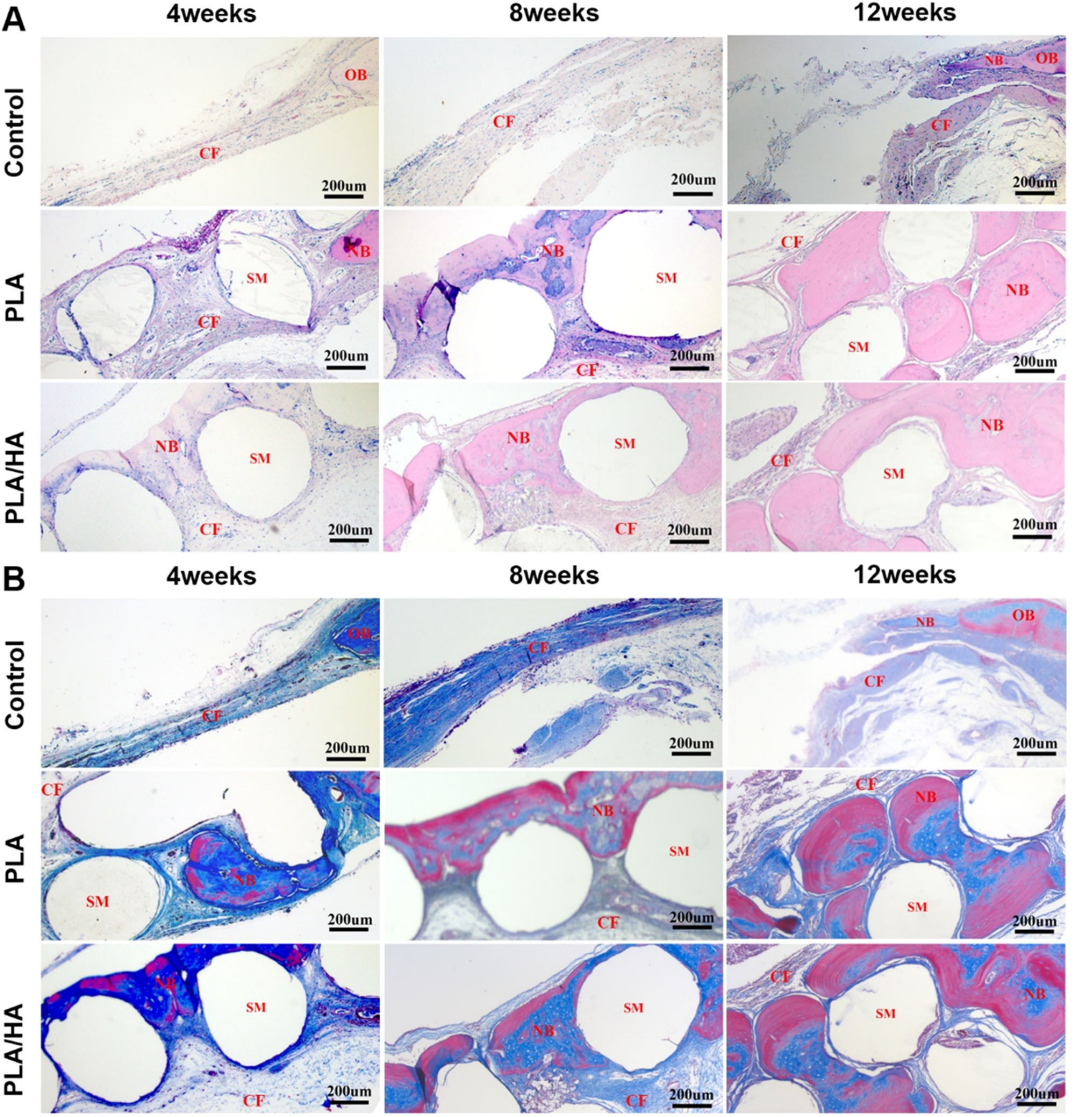
Immunohistochemical staining in the different groups at 4, 8, and 12 weeks; **A** Hematoxylin-eosin (H&E) and **B** Masson’s trichrome staining of tissue sections in different groups at 4, 8 and, 12 weeks after surgery. OB Original bone tissue, CF Collagen fiber, SM Scaffold materials, NB New bone tissue

HE and Masson staining at 4, 8 and, 12 weeks after surgery showed similar tissue reactions among all the groups throughout the study (Fig. 8A, B). At four weeks after treatment, compared with that in the implantation group, only a few collagen fibers formed in the control group. However, many collagen fibers were visible in the PLA and PLA/HA groups. The skull defect was filled with different amounts of new bone (12.22 ± 1.46% and 14.61 ± 1.71%), and the shape of the scaffold was visible. At 8 and 12 weeks after surgery, many collagen fibers were observed in the control group, but only a tiny amount of new bone formed at the defect edge. New bone tissue formed progressively in the PLA and PLA/HA groups (Table 3). Furthermore, in the PLA/HA group, the defect was filled with new bone at 12 weeks (46.17 ± 2.47%), with less scaffold material remaining compared to that in the PLA group. These results indicate that PLA/HA can act as an adequate bone substitute and promote bone regeneration in the defect area.

**Table. 3:** The quantitative evaluation of newly formed bone area percentage in each group by immunohistochemical staining

Groups	4 weeks (%)	8 weeks (%)	12 weeks (%)
Control	0.45 ± 0.11	0.92 ± 0.26	2.60 ± 0.58
PLA	12.22 ± 1.46	26.36 ± 2.68	39.60 ± 2.76
PLA/HA	14.61 ± 1.71	28.91 ± 2.15	46.17 ± 2.47

**Fig. 9:**
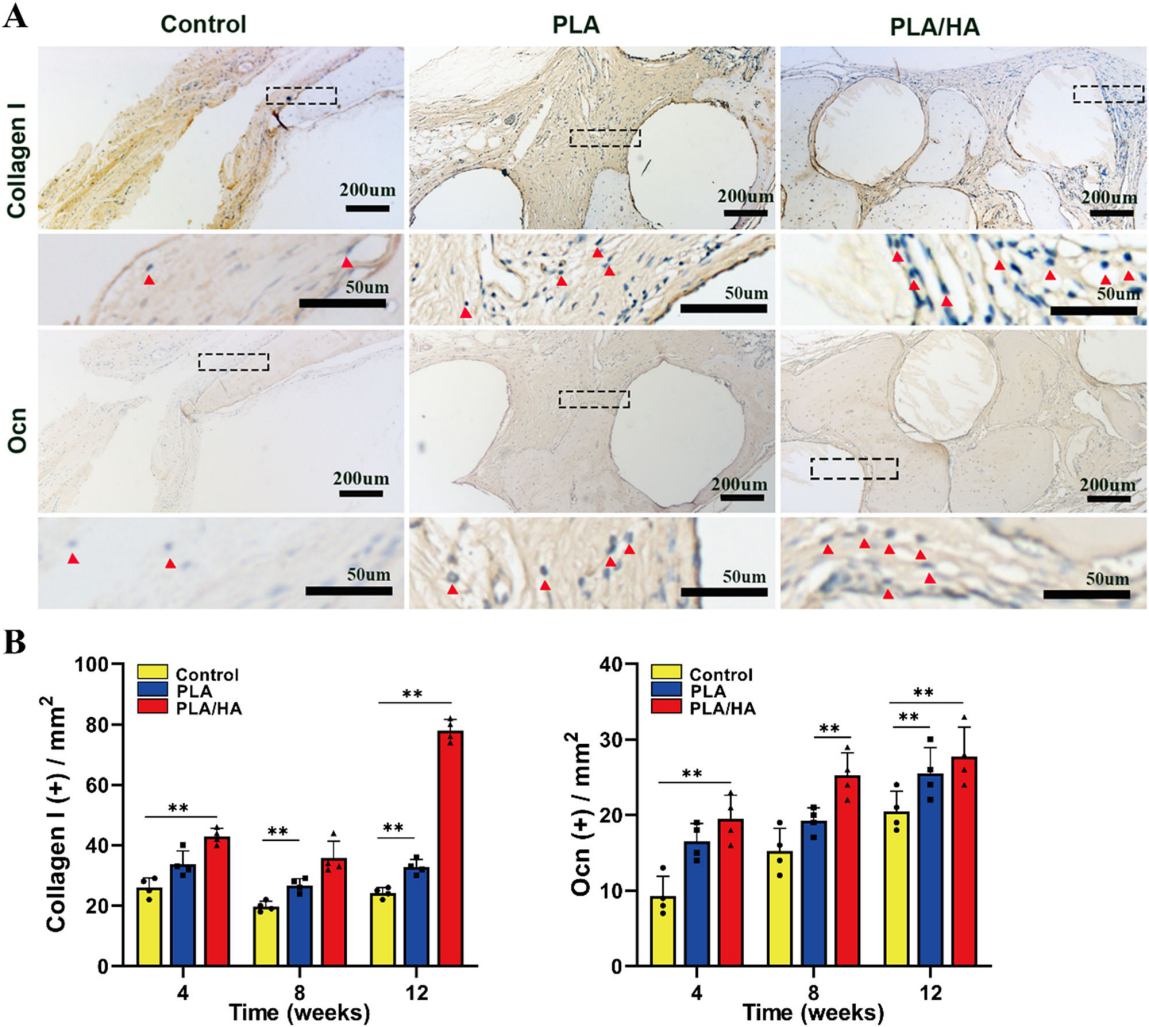
Osteoblasts in bone defect area after treatment; **A** Immunohistochemical staining of *Collagen I* and *OCN* in the different groups at 12 weeks. The black dotted boxes in the upper figures were enlarged in the lower figures. The red triangle indicates positively stained cells. **B** The quantitative assessment of the numbers of Collagen I and Ocn at 4, 8, and, 12 weeks after surgery. *p < 0.05; **p < 0.01. *n* = 4 per group per condition

Histological sections were examined with immunohistochemical staining for osteoblast markers at 12 weeks after treatment (*COLI* and *OCN*) to further assess osteogenic induction. *COLI* was positively expressed in both the PLA and PLA/HA groups (Fig. 9). The expression of *OCN* was evident in the newly formed bone tissue around the scaffold, and was barely found in the control group. These results revealed that the PLA/HA scaffolds with USCs significantly promoted osteogenic induction in the defect area.

## Discussion

Bone defects have become common in clinical practice, and the natural healing process is challenging because of the poor regenerative capacity and major defects. In this study, a 3D-printed PLA/HA composite scaffold containing USCs was successfully applied to a rat model of skull defects. The PLA/HA scaffold has the advantage of two kinds of scaffolds. The mechanical properties of the PLA/HA scaffold increased and the degradation rate improved,. In addition, the feasibility of combining scaffolds with USCs was verified in vitro. At 12 weeks after treatment, effective bone regeneration indicated the good therapeutic effect of the PLA/HA scaffold and USCs in tissue engineering.

Applying bone tissue engineering technology to treat bone defects, such as preparing 3D-printed scaffolds carrying stem cells, has been considered a new treatment strategy in recent years [14]. In a study on the treatment outcomes of custom-made bioceramic implants, Staffa et al. [27] found that most patients recovered well after surgery with few adverse consequences, demonstrating the osteogenic properties of HA. Another study showed that the mechanical properties of HA are not strong enough to be used in large-scale defects [28]. However, a single material does not have all the properties required for tissue engineering. Therefore, different biopolymers and minerals have been used to fabricate scaffolds with other properties [29]. Zimina et al. produced PLA/HA composites and showed their potential for complex tissue engineering and restoring maxillofacial defects [20]. Ideal scaffold materials for bone tissue engineering have increased biocompatibility, biodegradation, and mechanical strength; otherwise, it is difficult to achieve bone defect repair [30, 31]. Although PLA has good biocompatibility and degradation, it is still deficient in mechanical strength and bone conductivity. Therefore, HA was combined with PLA in this study, and the results further proved that the mechanical performance and properties of composite scaffolds are superior to those of the individual materials (Table 2).

By extruding thermoplastic materials through a heated metal nozzle, FDM fabrication produces various 3D designs [32]. For example, FDM-technology-printed porous polycaprolactone and hydroxyapatite (PCL/HA) scaffolds support cell adhesion and proliferation and prevent the adverse effects of inflammation on murine chondrocytes [33]. The physicochemical properties of the scaffold are crucial for the growth and differentiation of stem cells. The high porosity and interconnected pore structure of scaffolds are believed to be fundamental for bone regeneration in tissue engineering, and the optimum pore size ranges from 20 to 1500 μm. The size of the macropores of the scaffold is supposed to be > 350 μm because stem cells need to migrate to the defect site and mediate bone regeneration [28]. Different pore sizes can be easily acquired by changing printing parameters such as the nozzle diameter, movement speed, and extrusion pressure [34]. In our study, a PLA/HA composite scaffold was fabricated by FDM technology with pore sizes ranging from 300 to 500 μm. The distribution and adhesion behavior of USCs in the PLA/HA group was more notable than that in the other groups. Our results showed that the PLA/HA composite scaffold had better mechanical properties and cytocompatibility than the PLA-only scaffold (Fig. 4), which was consistent with the results from previous studies [20].

The mechanical properties of scaffolds can be increased by combining PLA with HA. However, even the maximum values of Young’s modulus and the ultimate deformation strength of the printed scaffolds are many times less than those of bone tissue; hence, printed scaffolds may not be suitable for weight-bearing applitions. In our study, printed scaffolds were not used as load-bearing materials but as osteoinductive biomaterials containing USCs to promote bone regeneration in the defect site. In previous studies, the biomaterials in scaffolds have played a crucial role in promoting bone regeneration. For instance, PLA/HA implantation in mice exhibited good tolerance and promoted widespread ingrowth of newly formed bone tissue in the implant pores [20]. Our in vitro results suggested that the PLA/HA composite scaffold induced pronounced osteogenic gene expression (Fig. 5), which indicated that the PLA/HA composite scaffold enhanced the osteogenic potential of USCs.

Although different materials have different abilities to repair bone defects, successful bone regeneration still requires stem cells that can differentiate into other types of cells. Stem cells such as bone marrow MSCs, adipose-derived stem cells, muscle-derived stem cells, and embryonic stem cells, have long been used in scaffolds for tissue engineering [35–38]. However, the clinical application of most stem cells is limited by various factors, such as limited sampling sites, insufficient donors, and poor cell homing. Similar to other adult stem cells, USCs have cell characteristics such as a specific growth pattern and differentiation, and these cells have been successfully used for the urinary system, nerve tissue, and skeletal muscle tissue injury [9–12]. Our work showed that USCs expressed the surface marker of bone marrow MSCs, and had notable cell proliferation and multiple differentiation potential (Fig. 1). In addition, USCs have been used to repair and reconstruct bone defects. For example, the combination of PLGA/CS scaffolds and USCs has potential in bone regeneration since these scaffolds can stimulate osteogenic differentiation in USCs and induce the ingrowth of blood vessels into scaffolds [39]. Many researchers have attempted to develop methods to induce USCs to the osteogenic lineage [40], but few studies have focused on combining USCs with scaffolds that are suitable for tissue engineering. Furthermore, the effects of PLA/HA composite scaffolds on the osteogenic differentiation of USCs have not been reported. Therefore, we fabricated a PLA/HA composite scaffold with FDM 3D printing technology. USCs were seeded onto scaffolds, which were subsequently transplanted into rats to analyze the osteogenic differentiation of USCs.

Our in vivo study showed that the PLA/HA scaffold containing USCs repaired bone defects at the macro and micro levels (Figs. 6–8). General observation and micro-CT images showed that the implantation group exhibited a better therapeutic effect than the control group at 12 weeks after treatment. The dimension of the newly formed bone was analyzed by micro-CT analysis and indicated that the PLA/HA scaffolds containing USCs had the best performance in reconstructing bone defects and the fastest scaffold degradation rate. Osteocalcin (*OCN*) plays a vital role in bone building and late osteogenesis. Compared with that in the other groups, more *OCN* was expressed in the PLA/HA group, indicating that the PLA/HA scaffold further promoted osteogenic differentiation in USCs. The PLA/HA scaffold containing USCs caused more marked bone regeneration in the skull defect model than the PLA scaffold with USCs.

There were several limitations to our study. Although USCs have been successfully seeded onto PLA/HA scaffolds and applied to bone defect repair, it is still unclear whether USCs can be widely used in other kinds of scaffolds. Additionally, we evaluated bone defect repair, but the weight-bearing capacity and mechanical properties of the newly formed bone in the defect area still have to be further tested. Finally, in this bone defect model, we did not analyze the mechanism of bone defect repair, which requires further investigation.

## Conclusion

The PLA/HA composite scaffold was prepared by 3D printing and exhibited excellent biocompatibility and mechanical intensity. The in vivo test results demonstrate that the PLA/HA scaffold containing USCs can promote the progressive regeneration of bone tissues in the defect area. These findings suggest that composite scaffolds inoculated with USCs are a reliable solution for bone tissue engineering.

## Data Availability

The datasets used and/or analyzed during the current study are available from the corresponding author on reasonable request.

## Abbreviations

**PLA**: Polylactic acid
**HA**: Hydroxyapatite
**USCs**: Urine-derived stem cells
**MSCs**: Mesenchymal stromal cells
**3D**: Three-dimensional
**CAD**: Computer-aided design
**FDM**: Fused-deposition modeling
**PBS**: Phosphate buffered saline
**FBS**: Fetal bovine serum
**PDGF**: Platelet-derived growth factor
**TGF-β**: Transforming growth factor-β
**DMEM**: Dulbecco’s modified Eagle’s medium
**EDS**: Energy-dispersive X-ray spectroscopy
**SEM**: Scanning electron microscopy
**IACUC**: Institutional Animal Care and Use Committee
